# Enhancing RNA Capture Efficiency in Spatial Transcriptomics: A Review of Innovative Technologies and Strategies

**DOI:** 10.3390/ijms262211076

**Published:** 2025-11-16

**Authors:** Qinyu Ge, Yuqi Sheng, Yuting Shan, Yuwei Yang, Haohan Jiang, Ruyue Wang

**Affiliations:** State Key Laboratory of Digital Medical Engineering, School of Biological Science and Medical Engineering, Southeast University, Nanjing 210096, China; sheng_yuqi@163.com (Y.S.); shanyt0504@163.com (Y.S.); yoy@seu.edu.cn (Y.Y.); 18851003579@163.com (H.J.); wangruyuede@163.com (R.W.)

**Keywords:** spatial transcriptomics, RNA capture efficiency, microfluidic chip, nanomaterials, FFPE sample, computational prediction

## Abstract

Spatial transcriptomics technology represents a groundbreaking advancement in the life sciences, enabling the analysis of gene expression patterns within their native spatial context. However, inefficiencies in RNA capture from tissue samples have historically limited its effective application. This article presents a systematic review of innovative technologies and strategies that have enhanced the efficiency of spatial transcriptome RNA capture in recent years. These strategies include nanomaterial-enhanced capture, optimization of microfluidic chips, advancements in molecular biology techniques, and computationally assisted prediction methods, among others. Through a comparative analysis of cutting-edge technologies such as Decoder-seq, Stereo-seq V2, MAGIC-seq, and MSN-seq, this article summarizes progress made in addressing challenges related to RNA diffusion, probe density, and tissue processing. Particular emphasis is placed on optimization approaches for formalin-fixed paraffin-embedded (FFPE) clinical samples and computational prediction methodologies that integrate artificial intelligence. These innovations provide valuable references for future technological development. The objective of this review is to provide researchers with a comprehensive understanding of how to enhance spatial transcriptome capture efficiency while promoting the utility of this technology in both basic research and clinical applications.

## 1. Introduction

Recently, spatial transcriptomics technology has rapidly become a prominent research topic in the life sciences and related fields [1,2,3]. By combining gene expression data with spatial distribution in situ, it offers novel insights into areas such as developmental biology [4,5], neuroscience [6,7,8], and oncology [9,10,11,12]. However, practical applications of this technology face several challenges, with one of the most significant being the low RNA capture efficiency in tissue samples. This efficiency directly impacts detection sensitivity and data reliability, which is especially critical for studying low-expression genes and rare cell types [13,14].

RNA capture efficiency in spatial transcriptomics can be defined as the proportion of RNA molecules released from tissue sections that are successfully captured. Which can be measured by the number of UMIs (UMIs/μm^2^) or molecules (molecules/μm^2^) captured per unit area. The RNA capture efficiency of current mainstream spatial transcriptomics techniques remains generally unsatisfactory. For example, Decoder-seq technology, developed by Professor Yang’s team at Xiamen University, significantly improves capture performance through a microfluidic-assisted orthogonal coding strategy [15,16]. However, it still achieves a capture efficiency of only 20% to 30%. Although this is a leading figure among similar technologies, it means that over 70% of the target RNA is not effectively captured, resulting in the loss of critical biological signals. This limitation is particularly pronounced in the detection of low-expression genes, making it challenging to fully reveal the true transcriptional state of the tissue.

The factors influencing RNA capture efficiency are complex and multifaceted. These include inherent limitations of the underlying technical principles, the effects of tissue processing methods, probe design strategies, and the optimization of experimental conditions. For example, methods based on spatial barcode arrays provide unbiased analysis capabilities but are constrained by probe density and affinity [17,18,19,20]. Fluorescence in situ hybridization (FISH) methods offer relatively high sensitivity but can only detect predetermined genes [21,22,23,24,25]. Additionally, commonly used FFPE samples in clinical practice pose further challenges to capture efficiency due to severe RNA degradation [26,27,28,29].

This article aims to systematically identify the key factors influencing RNA capture efficiency in spatial transcriptomics technology. It reviews the latest technological advancements, evaluates the effects of various strategies on efficiency improvement, and suggests directions for future technological development. A comprehensive analysis of solutions to this critical technical bottleneck is expected to facilitate the transformation of spatial transcriptomics from a technical platform into a tool for biological discovery.

## 2. Key Factors Influencing the Efficiency of Spatial Transcriptome RNA Capture

The RNA capture efficiency of spatial transcriptomics technology is influenced by multiple factors that collectively determine its overall performance quality. A thorough understanding of these factors is essential for developing effective capture strategies.

### 2.1. Inherent Limitations of the Technical Principle

**Figure 1:**
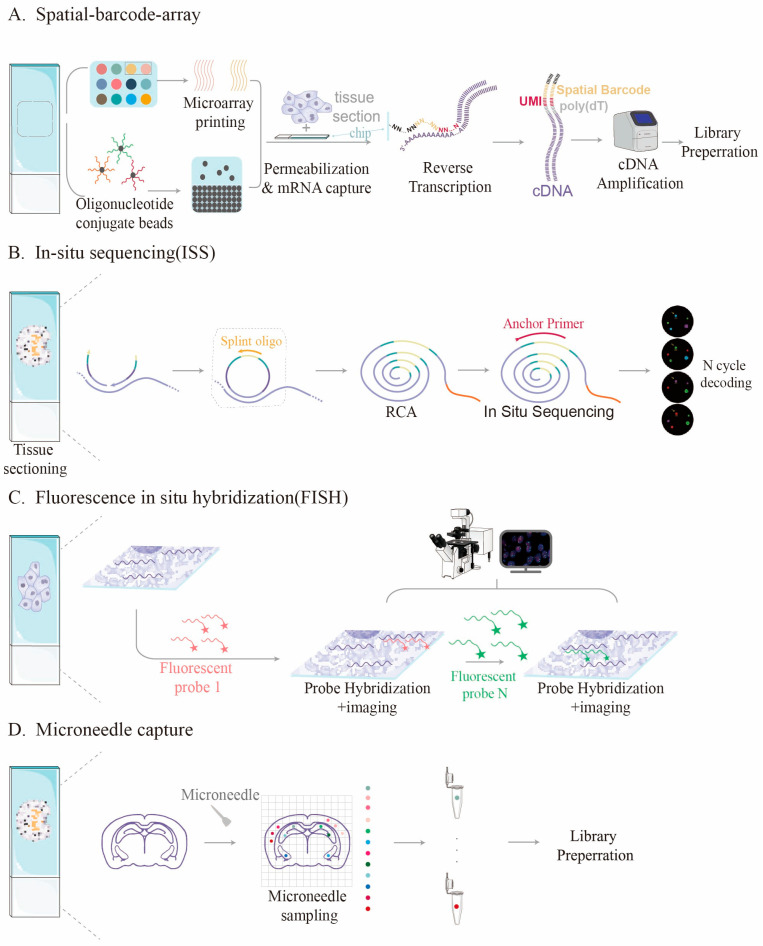
Different principle of various spatial transcriptome methods. (**A**) Detection process of spatial barcode-based method, dots of different colors represent distinct encoded capture probes; (**B**) In situ sequencing method; (**C**) Fluorescence in situ hybridization-based method; (**D**) Method for sample captured with microneedles, dots of different colors represent distinct samples captured by the microneedle.

Different spatial transcriptomics techniques are based on various working principles, each with inherent limitations in capture efficiency. The main principles of these spatial transcriptomics methods are illustrated in Figure 1. Methods based on spatial barcode arrays, such as 10 × Visium [1] and Stereo-seq [29,30], capture mRNA released from tissues by affixing a large number of oligonucleotide probes to the surface of a chip. However, their efficiency is constrained by the density and affinity of the probes. Research by Professor Yang’s team indicates that the density of DNA probes on traditional planar substrates is limited, making it difficult to comprehensively capture high-density RNA molecules in tissues [15]. Conversely, methods based on in situ sequencing are limited by imaging resolution and detection sensitivity, which complicates accurate quantification of expression levels [31,32]. Table 1 shows a comparison of capture efficiencies among different spatial transcriptomics methods.

### 2.2. The Impact of Organizational Processing Methods

The organizational processing flow significantly impacts the efficiency of RNA capture. Although fresh-frozen tissues generally maintain high RNA integrity, their preparation and storage require stringent conditions [41,42]. The capture of sliced RNA can be influenced by embedding, sectioning, and permeation. When preparing tissue sections for spatial transcriptomics studies, thickness is critical [43]. If the sections are too thick, they become difficult to penetrate, resulting in greater RNA loss. Conversely, if the sections are too thin, obtaining complete cells becomes challenging, complicating subsequent data analysis. Tissue section penetration is a highly precise task, and timing must be carefully controlled. If the permeation time is too long or too short, it will adversely affect RNA capture. Incomplete penetration prevents the chip sites from capturing mRNA, which can cause random drift. Excessive permeation can lead to the release of large amounts of mRNA, which may be captured by adjacent sites, thereby compromising data accuracy [44,45]. Furthermore, all these steps may contribute to RNA degradation, negatively impacting capture efficiency [46].

FFPE samples are the most common in clinical practice and are easy to preserve; however, nucleic acid cross-linking and fragmentation caused by formaldehyde fixation significantly reduce capture efficiency [47,48]. Research by BGI revealed that the DV200 value of RNA in FFPE samples, an indicator of RNA degradation, can be as low as 18, which greatly increases the difficulty of efficient capture [49,50].

To address this issue, the Stereo-seq V2 technology was developed with dedicated steps for deparaffinization, rehydration, and cross-linking. These steps significantly improve compatibility with FFPE samples. Additionally, variables such as tissue section thickness, fixation time, and pretreatment methods can affect RNA accessibility and require optimization for different tissue types [29].

### 2.3. Probe Design and Capture Strategy

The molecular basis of probe design determines capture efficiency. The traditional poly(T)-primed strategy primarily targets mRNA with poly(A) tails and cannot capture important transcripts, such as non-coding RNAs. Furthermore, this approach performs poorly with FFPE samples due to RNA degradation caused by the fixation process. Stereo-seq V2 innovates by using random hexamer primers (6N) instead of poly(T) primers to achieve unbiased capture of the entire transcriptome [29,46]. This enhances mRNA capture efficiency and enables the detection of non-coding RNAs and pathogen transcriptomes.

Decoder-seq increased the density of barcode modifications by approximately tenfold by constructing three-dimensional, tree-like nanoscale substrates. This significantly increased the number of capture sites per unit area, thereby enhancing mRNA capture efficiency [15,16]. This combination of physical optimization and chemical design represents an important advancement in improving capture efficiency.

### 2.4. RNA Quality and Experimental Conditions

The quality of the input RNA directly impacts the final capture efficiency. Even with the most advanced techniques, achieving optimal results is challenging when RNA samples have undergone severe degradation [51,52]. Additionally, experimental conditions such as permeation time, hybridization temperature, and enzyme activity can influence capture efficiency. Therefore, the experimental protocol must be optimized for sample systems from different sources to ensure efficient RNA release and capture while preserving tissue morphology.

In conclusion, the efficiency of RNA capture from the spatial transcriptome is influenced by multiple factors, including technical principles, sample processing, probe design, and experimental conditions. Addressing this bottleneck requires multidisciplinary collaboration and comprehensive optimization across various fields, such as materials science, molecular biology, and computational analysis.

## 3. Innovative Technologies and Strategies for Enhancing Capture Efficiency

To address the issue of low efficiency in capturing spatial transcriptome RNA, multiple research teams have proposed innovative solutions from various perspectives. These technical strategies have significantly enhanced the performance and practicality of spatial transcriptomics (Table 2).

### 3.1. Technological Innovation Based on Nanomaterials and Microfluidic Chips

**Figure 2:**
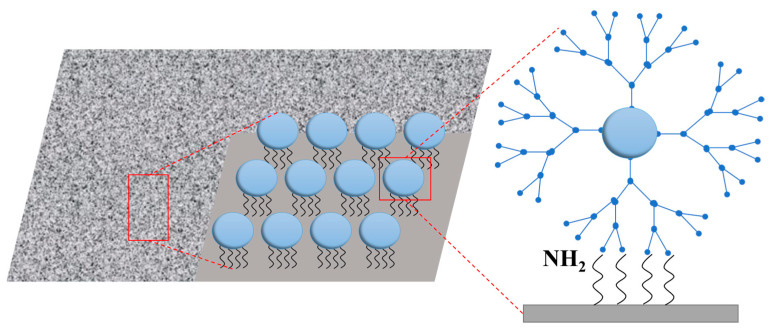
Strategy to enhance RNA capture efficiency using high-density spatial barcode arrays on three-dimensional nanostructured substrates.

Nanomaterial reinforcement is an effective method to increase probe density. Professor Yang’s team at Xiamen University developed Decoder-seq technology, which utilizes dendrimer DNA nanostructures to create high-density spatial barcode arrays on three-dimensional nanoscale substrates [15,16]. This approach increases the density of DNA probes by approximately tenfold (Figure 2). The design significantly enhances the number of capture sites per unit area, enabling detection sensitivity to reach 40.1 mRNA molecules per μm^2^, which is substantially higher than that of comparable methods. This high sensitivity allowed the team to detect the low-expressed olfactory receptor gene (*Olfr*) in the olfactory bulb of mice and to discover its unique layered distribution pattern.

**Figure 3:**
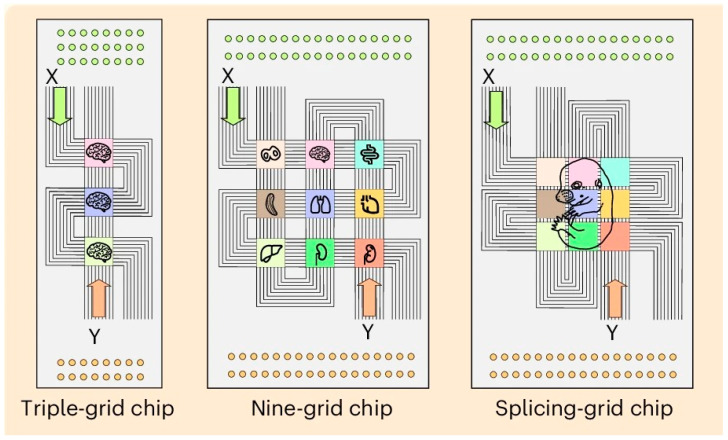
Innovation using a splicing chip. Adapted with from Zhu [53]. Efficient and cost-effective RNA capture from large-area tissue sections can be achieved through the innovative combination of microfluidic barcoding chip.

MAGIC-seq technology, developed by Zhao’s team at the Institute of Zoology, Chinese Academy of Sciences, enables high-throughput, wide-field spatial transcriptome analysis through a grid-based microfluidic chip design [53]. The innovation of this technology lies in the concept of the “splicing chip” (Figure 3). By adjusting the grid spacing and employing multi-round encoding technology, multiple capture grids can be seamlessly combined to expand the capture area to approximately 3.5 cm^2^ without compromising resolution. This design significantly reduces chip preparation costs to about $0.11/mm^2^, minimizes batch effects, and is well-suited for large-scale sample studies. In comparison, the preparation cost of 10 × Visium HD and Decoder-seq was about $5/mm^2^ and $0.55/mm^2,^ respectively. In addition, DBiT-Seq increases tissue capture efficiency by 30% and reduces coding costs to about $0.50/mm^2^ by using PDMS microfluidic vertical cross-microchannel coding. The cost comparison of different platforms can be seen in Table 3.

**Table 2:** Comparison of innovative strategies for improving RNA capture efficiency.

Innovation Strategy	Representative Technology	Mechanism/Principle	Efficiency Improvement Effect
3D nanostructured substrate	Decoder-seq [15]	Increase the density and accessibility of probes	The sensitivity has been increased by approximately ten times
Grid-based microfluidic chip	MAGIC-seq [53]	Expand the capture area and reduce the batch effect	The detection throughput has increased by 8 times
Random primer capture	Stereo-seq v2 [29]	Unbiased capture of the whole genome	23,459 genes outside the Visium probe set were detected
Microneedle targeted sampling	MSN-seq [40]	Precisely locate the cell population	Suitable for precious samples
Computational prediction compensation	KAFSTExp [54]	Predict expression from pathological images	Reduce reliance on experimental capture

### 3.2. Innovation in Molecular Biology Methods

At this stage, innovations primarily focus on optimizing primer design and library construction strategies. The most significant breakthrough in Stereo-seq V2 is the use of random primers (6N) instead of traditional poly(T) primers. This advancement allows the technology to capture the entire transcriptome impartially, including mRNA, non-coding RNA, and pathogen RNA, making it especially suitable for analyzing FFPE samples. The 6N sequence at the end of the random primers simultaneously serves as a unique molecular identifier (UMI), addressing the issue of PCR amplification bias.

**Figure 4:**
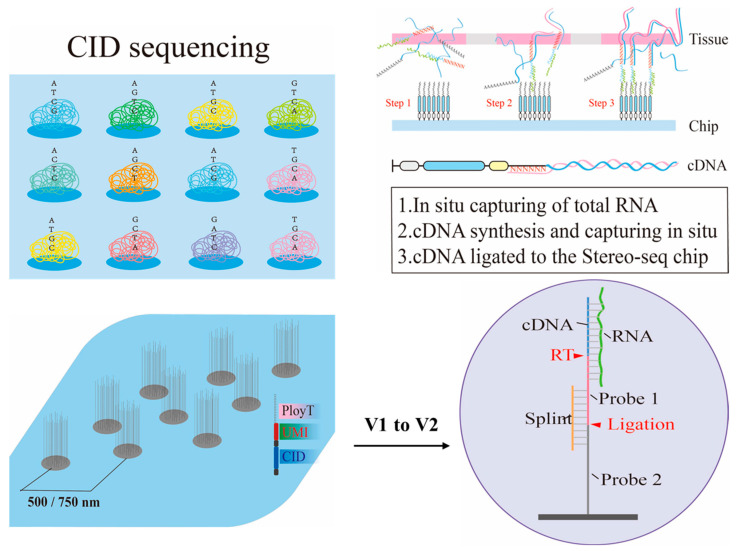
Strategy for enhancing capture efficiency using molecular biology methods. Nanoballs and random primers (6N) to improve RNA capture efficiency in Stereo-seq-based method.

As seen in Figure 4 Stereo-seq uses a DNA nanosphere (DNB) array to achieve a spatial resolution of 500 nm and an ultra-large field of view of 13 cm^2^. Each DNB carries a unique spatial barcode with a probe density of up to 18,000 per μm^2^, enabling gene capture at the single-cell level with a similar efficiency to that of single-cell RNA sequencing. Compared with version V1, Stereo-seq V2 detected 44,773 genes in similar mouse brain areas, an increase of 14,643 genes, including 3895 non-coding RNAs. When compared with 10 × Visium (FFPE), Stereo-seq V2 detected significantly more genes in mouse brain tissue experiments, and 23,459 genes found that were detected by Stereo-seq V2 solely. Additionally, the use of random primers enables reads to uniformly cover the entire gene length, regardless of GC content. This reduces the molecular diffusion distance (LWHM, left-width at half-maximum) by 50%, significantly enhancing spatial resolution [29].

The 10 × Visium HD employs three pairs of probes to target a single gene, utilizing an RNA template ligation mechanism to increase the gene detection rate by over 30% [55]. It eliminates the gaps between the traditional 55 µm spots and increases tissue coverage from 60% to 100%. In mouse intestinal samples, this technique can clearly distinguish gene expression gradients at different levels of intestinal villi [33]. Furthermore, this design significantly reduces non-specific background noise by enhancing the likelihood that the probe will bind specifically to the target RNA. MERFISH 2.0 introduces three key optimizations: (1) RNA anchoring technology, which ensures the co-localization of degradation fragments; (2) optimization of the encoded probe structure to improve binding efficiency; and (3) a multi-round fluorescence readout probe design that increases the signal-to-noise ratio threefold [56]. In mouse mammary FFPE samples, this technique can detect nearly ten times more transcripts than traditional methods [57].

Unlike traditional poly(A) capture methods, which exhibit a 3′ end bias, Well-ST-seq employs a high-density probe array generated by a microfluidic chip to achieve uniform, full-length transcript coverage. This approach enhances the sensitivity of detecting variable splicing events by 40% [58]. This design is especially well-suited for analyzing fusion genes and isoforms.

The Xenium platform improves the design of poly(A) probes by incorporating sites that are tolerant of 3′ end degradation and by integrating in situ reverse transcription technology to minimize RNA diffusion [19,59]. These optimizations enable capture efficiencies of 1.5–2.0 UMIs/μm^2^ in FFPE tissue samples, representing a 50% improvement on the Visium platform. The Seq-Scope platform uses photolithography to create high-density probe arrays (10^6^ probes per μm^2^) and employs a partitioned capture strategy combined with joint decoding, enabling the detection of 1800–2200 genes per cell in mouse brain tissue samples while maintaining lower data sparsity.

We developed MSN-seq (Musashi stainless steel needle-based sequencing technology) technology in a previous study, which employs a unique approach by combining microneedle sampling with an enhanced Smart-seq2 process. This technology precisely samples target tissue using a Musashi stainless steel needle (MSN) and is especially suitable for research involving small-scale cell populations. Although this targeted method reduces throughput, it efficiently captures specific regions of interest by minimizing RNA loss from non-target areas.

### 3.3. Computing and Artificial Intelligence-Assisted Strategies

Since there are physical limitations to improving capture efficiency at the experimental level, computational and artificial intelligence methods offer alternative approaches to compensate for insufficient capture efficiency.

Researchers have developed biological tools based on deconvolution algorithms to address the inherent limitations of spatial transcriptomics data, including low capture efficiency, limited resolution, and signal diffusion at neighboring sites. These algorithms typically combine high-resolution single-cell RNA sequencing (scRNA-seq) reference data with spatial transcriptomics profiles to infer the proportion and expression characteristics of each cell type at each spatial location. This computational approach decomposes the mixed signals and reconstructs spatial expression patterns at the cellular level, thereby partially compensating for low capture efficiency.

Representative tools include RCTD (Robust Cell Type Decomposition) and SpatialDWLS, which use weighted linear regression to estimate cell-type proportions within each spatial spot [60,61]. Stereoscope and Cell2location adopt Bayesian generative models to statistically model count variability and noise, providing robust deconvolution results even for low-quality data [62,63]. Tangram employs a deep learning-based optimization framework to map single-cell gene expression onto spatial coordinates, effectively restoring cell-level resolution. More recent methods, such as DSTG, GraphST, and SpaGCN, further incorporate graph neural networks (GNNs) to account for spatial neighborhood information and improve signal smoothing [64,65,66].

**Figure 5:**
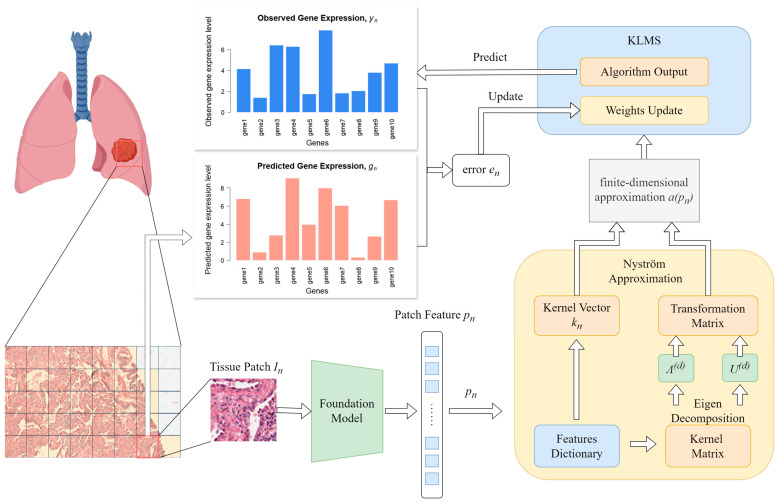
Computing and artificial intelligence-assisted strategies for enhancing capture efficiency. The figure shows that KAFSTExp employs computational methods to develop a predictive model capable of forecasting gene expression in cells directly from HE-stained images.

The KAFSTExp framework, developed by Xie’s team at the University of Electronic Science and Technology of China, innovatively predicts spatial gene expression from traditional hematoxylin and eosin (H&E)-stained pathological images (Figure 5). This method integrates the basic pathological image model UNI with Kernel Adaptive Filtering and employs the Nystrom approximation to achieve model sparsity and computational acceleration [54].

KAFSTExp performed exceptionally well in benchmark tests across ten types of cancer, with the average Pearson correlation coefficient (PCC) increasing by 19.80%. By bypassing the physical limitations of RNA capture, this method ‘predicts’ gene expression through computational means, offering an economical and efficient alternative for resource-limited scenarios. KAFSTExp demonstrates outstanding predictive performance for key cancer-related genes of high clinical significance, highlighting its potential value in tumor molecular typing, prognosis assessment, and the development of personalized treatment strategies.

In addition, the SpaIM model combines the ‘content’ of single-cell RNA-seq data with the ‘style’ of the spatial transcriptome using a style transfer algorithm. Tests on 53 datasets have shown that the sensitivity of differential gene detection has increased by 40% [67]. This method is especially effective at recovering signals from samples with low sequencing depth. The SpatialGlue graph neural network employs a dual attention mechanism to integrate multimodal data, significantly reducing the false positive rate caused by molecular diffusion in mouse hippocampal samples [68,69]. Through a multi-level attention mechanism encompassing sector, cell, and molecular levels, the batch effects in breast cancer sample sections were effectively removed [70]. The spatial associations related to macrophages and the tumor invasion frontier were accurately identified in the analysis of the breast cancer microenvironment.

Open-ST uses a capture efficiency correction module to integrate multi-platform spatial transcriptomics data and establish a unified predictive framework. This enhances the sensitivity with which low-abundance genes are detected in low-efficiency samples by over 30% [71]. The platform supports various data types, including Visium, Stereo-seq and MERFISH. The SpaQC quality control tool is designed for imaging-based spatial transcriptomics platforms and improves the robustness of MERFISH datasets by reducing sparsity by 15–20%. This is achieved through the systematic evaluation of signal intensity and spatial distribution, as well as the correction of uneven capture efficiency arising from probe hybridization variability.

Collectively, these bioinformatics approaches significantly mitigate the effects of low RNA capture efficiency and molecular diffusion by reconstructing high-fidelity spatial gene expression patterns. Although they cannot physically increase capture efficiency, they serve as powerful computational tools that enhance spatial resolution and improve the interpretability of spatial transcriptomic data.

### 3.4. Imaging Spatial Transcriptome Platform

MERFISH 2.0 uses multiple orthogonal coding and in situ signal amplification strategies with probes that target between 1000 and 5000 genes. It achieves a capture efficiency of 3.0 to 5.0 molecules/μm^2^ in mouse hippocampal tissue with a lateral full width at half maximum (LFWM) of just 5 to 8 μm, demonstrating its superior spatial resolution. However, its performance in FFPE samples is limited, requiring a DV200 value of at least 50%. By contrast, STARmap Plus enhances the in situ reverse transcription and rolling circle amplification processes, enabling the amplification of RNA signals by a factor of 100 to 1000. It excels at detecting low-abundance RNAs in synaptic clefts within brain tissue and can profile 1200–1500 genes per cell with lower data sparsity [72].

SABER-FISH reduces dependence on probe concentration through the rational design of signal amplifiers, improving the capture efficiency of target genes in FFPE samples by 40%. Reliable detection can be achieved when DV200 is equal to or greater than 30%, making this method ideal for the spatial profiling of target genes in clinical specimens [73]. ExSeq combines tissue clearing with in situ sequencing technology to address the issue of limited penetration in thick tissues. It maintains a capture efficiency of 2.5–3.0 molecules/μm^2^ in 50 μm thick mouse brain sections, with low spatial diffusion [31].

### 3.5. Comprehensive Optimization Plan

The Spatiotemporal Transcriptome FFPE V1.1 product solution represents the latest advancement in comprehensive optimization [74,75]. This solution enhances capture efficiency through four core innovations: (1) gene capture efficiency increased by 50–200%; (2) optimization of signal diffusion both within and outside the tissue; (3) a 100% increase in data utilization rate; and (4) support for mixed testing of fresh frozen (FF) and FFPE samples. Notably, this scheme significantly enhances the capture efficiency of FFPE samples by optimizing probe design and biochemical reaction conditions. In test samples, including lung, liver, and lymph node cancer tissues, cDNA yield increased by approximately 100%, and the utilization rate of effective data improved by about 40%. This comprehensive optimization strategy exemplifies the evolution of spatial transcriptomics technology from isolated breakthroughs to integrated systems. Penetration time is a critical parameter influencing capture efficiency. Spatiotemporal Transcriptome FF V1.3 shortens the optimal penetration time window from 15–30 min to 5–10 min by monitoring the fluorescence signal in real time, while maintaining a high gene capture rate, the median number of genes at the Bin20 level increases by 149% [29].

The EX-ST integrates tissue expansion technology with a dual-probe design to overcome the resolution limitations of traditional spatial capture arrays. Its primary function is to expand gel-embedded tissues by at least 2.5-fold, enabling high-resolution RNA capture [76]. When adapted to the Visium platform, EX-ST improves spatial resolution from 55 µm to 20 µm and significantly increases the number of captured UMIs. Array-Seq employs established oligonucleotide microarray technology combined with integrated probe assembly and spatial barcode design to deliver efficient, cost-effective spatial transcriptome profiling with broad sample coverage.

**Table 3:** Performance Metrics of Representative Spatial Transcriptomics Technology Platforms [15,19,29,35,36,37,46,50,53,77] ^‡^.

Technique Platform	Nominal Resolution	Proxy for Capture Efficiency	Gene Detected per Unit	Sparsity	Diffusion Metric	Data Usage (Valid Barcodes/Reads)	FFPE Compatibility	Cost of per Unit	Tissue Sample
Sequencing-based technology									
Stereo-seq V2	Subcellular	2–5 UMIs/μm^2^	42,440 genes probed	Low-Medium	Low	90–95% (Valid Reads)	DV200 ≥ 18%	~$35/mm^2^	Mouse brain FFPE.
Visium HD	2 μm features	248–670 UMI per 8 μm bin	300–400 genes per 8 μm bin	Medium	Medium	85–90% (Valid barcodes)	DV200 ≥ 30%	~$5/mm^2^	Pancreatic cancer FFPE
Decoder-seq	Flexible	40.1 UMIs per μm^2^ for 15-μm-spot and 35,720 UMIs per spot for 50-μm-spot	14.7 genes per μm^2^ for 15-μm-spot and 7436 genes per spot	Low	Low	85–90% (Valid barcodes)	DV200 ≥ 35%	~$0.55/mm^2^	Mouse olfactory bulbs
MAGIC-seq	Flexible	High 155,630 UMIs per spot (50-μm-spot)	5576 genes per spot (50-μm-spot)	Low	Low	86–91% (Valid reads)	DV200 ≥ 40%	~$0.11/mm^2^	Mouse olfactory bulb
DBiT-seq	10–50 μm pixels	Medium ~3000 UMIs/spot for 10 μm pixel; ~10,000 UMIs/spot for 50 μm pixel	~2068 genes per 10 μm pixel; ~4000 genes per 50 μm pixel	Medium	Medium	~87% (Valid barcode)	DV200 ≥ 30%	~$10/mm^2^	Mouse embryo
Imaging-based technology									
MERFISH 2.0	Subcellular	Up to 1 × 10^9^ transcripts per 1 cm^2^	100–1000+ genes probed	Low	Ultralow	90–95% (Valid reads)	DV200 must be ≥50%	NA	Human fibroblast cells
Xenium	Subcellular	1–5 UMIs/μm^2^ (panel-dependent)	1088 Median transcripts per cell (Panel-dependent)	Medium	Low	92–96% (Valid barcodes)	DV200 ≥ 25%	NA	Mouse FF Brain Hemisphere
Stereo-cell	Single-cell	6101 UMIs/cell	2139 genes/cell	Medium-Low	Low	88–93% (Valid reads)	DV200 ≥ 18%	NA	human PBMCs.

^‡^: The number of genes identified and the capture efficiency vary significantly across tissue types and among researchers. This table provides only a single representative data point for reference purposes. The DV200 value for FFPE sample compatibility is based on descriptions from related articles and website data. It is provided for reference purposes only. Data sparsity is typically represented by the fraction of non-zero features, with lower values indicating greater sparsity. Diffusion is commonly measured by the LWHM. Due to limited data availability, diffusion and sparsity is only categorized into high, medium and low levels. To ensure clarity and completeness, all references have been consolidated and are cited in the title of the Table, accounting for variations in the sources of different performance parameters on the same platform.

## 4. Comparison of the Performance of Various Technical Platforms in Terms of Capture Efficiency

As spatial transcriptomics technology has rapidly advanced, differences in capture efficiency among various platforms have become increasingly apparent. This system compares the performance metrics of different technologies to assist researchers in selecting the most suitable platform for their specific needs.

### 4.1. Comparison of Sensitivity and Resolution

Sensitivity is a key metric used to evaluate the efficiency of RNA capture, directly reflecting a technology’s ability to detect genes with low expression levels. Among current mainstream technologies, Decoder-seq stands out with its 3D tree-like nano substrate design, achieving a detection sensitivity of up to 40.1 mRNA molecules per μm^2^ and approaching near-single-cell resolution (15 μm). This high level of sensitivity enables the technology to identify mRNA enriched in the dendrites of neurons in the mouse hippocampus and to construct spatial single-cell maps. The DBiT-seq platform employs microfluidic channels to facilitate orthogonal spatial barcoding, offering adjustable spatial resolutions ranging from 10 to 50 µm and a moderate capture efficiency of approximately 10%. A distinguishing feature of DBiT-seq is its compatibility with diverse tissue types and molecular assays, including the simultaneous profiling of mRNA and proteins, which enhances its utility in multi-omics research [77].

Stereo-seq V2 achieves a balance between single-cell resolution and a centimeter-scale field of view on a 500 nm pitch chip by utilizing a random primer strategy. This technology detects nearly twice as many genes in mouse brain tissue studies compared to the V1 version, excelling particularly in capturing intron reads and detecting non-coding RNA. Its capability to provide full transcriptome coverage offers a distinct advantage for studying transcriptional regulation and pathogen–host interactions. Recently, Stereo-cell technology has achieved cell-level spatial transcriptomic profiling by integrating high-density DNA nanoball patterning with optimized tissue imaging and cell segmentation pipelines. By combining molecular barcoding with morphological cell boundary detection, it enables true single-cell spatial resolution and enhanced RNA capture efficiency. This makes Stereo-cell particularly valuable for characterizing fine-grained tumor microenvironment architecture and intercellular communication networks [50].

MAGIC-seq technology achieves an optimal balance between sensitivity and field of view. Its innovative design provides a capture area of approximately 3.5 cm^2^ while maintaining near single-cell resolution. This technology has demonstrated robust performance in three-dimensional brain reconstruction across multiple developmental stages in mice, successfully generating high-quality spatial transcriptome maps from the embryonic period onward.

10 × Visium HD has been introduced as an advanced iteration of the conventional Visium platform, providing a substantial improvement in spatial resolution and sensitivity. Unlike the standard Visium array with 55 µm capture spots, Visium HD employs a high-density spatial barcoding scheme that achieves 2 µm resolution, enabling near single-cell or even subcellular transcriptome profiling. In benchmark studies using mouse brain and human colorectal cancer tissues, Visium HD demonstrated markedly improved sensitivity, detecting over 15,000 genes per tissue section and capturing low-abundance transcripts that were previously undetectable with standard Visium [19]. The 10 × Xenium platform is a complementary solution centered on high-plex in situ hybridization chemistry. It enables the simultaneous detection of thousands of transcripts with subcellular resolution. Although the platform is designed to target a specific gene panel rather than the entire transcriptome, its hybridization-based approach ensures high capture efficiency (over 80%) and strong reproducibility. This makes it particularly well-suited for FFPE and clinical specimens [78].

In addition to the representative methods mentioned above, a variety of distinct spatial transcriptomics technologies have been developed. Table 3 summarizes their key performance metrics, including capture efficiency, the number of detected genes, and data sparsity.

### 4.2. Performance in FFPE Samples

The efficiency of RNA capture from FFPE samples is a crucial measure of the technology’s practicality. Stereo-seq V2 represents a significant advancement in FFPE sample compatibility. Even with severely degraded samples exhibiting a DV200 value as low as 18, a cDNA yield of 16.85 ng/mm^2^ can be achieved [79]. The technology was tested on ten triple-negative breast cancer samples preserved for over nine years, demonstrating its robustness across samples with varying degrees of degradation.

BGI’s Spatiotemporal Transcriptome FFPE V1.1 product solution has been specifically optimized for FFPE samples, achieving significant improvements across multiple areas. The protocol supports high-throughput analysis of tissue microarray (TMA) wax blocks, enabling FFPE samples from various puncture sites to be combined on a single chip. This greatly enhances the efficiency of retrospective clinical studies. Additionally, its innovative mixed-test compatibility allows FF and FFPE samples to be analyzed simultaneously on the same chip, reducing batch effects.

The 10 × Genomics Visium CytAssist system prevents RNA loss during the conventional patching process by transferring transcriptome analytes from standard slides to capture chips. Clinical data demonstrate that this technology doubles the effective utilization rate of FFPE samples, enabling retrospective analysis after H&E staining [80].

### 4.3. Analysis of Applicable Technical Scenarios

Technologies with varying capture efficiencies are best suited to specific application scenarios. For instance, research requiring high-sensitivity detection of low-expressed genes, such as receptor distribution studies in neuroscience, benefits from high-sensitivity platforms like Decoder-seq. Conversely, when processing a large number of FFPE samples for extensive clinical cohort studies, high-throughput solutions like those offered by BGI and Stereo-seq V2 are more appropriate.

Targeted technologies, such as MSN-seq, offer unique advantages for studying precious samples or specific cell populations. When experimental conditions are limited or rapid screening is necessary, computational prediction methods like KAFSTExp provide a practical alternative. To choose the most appropriate technical platform, researchers should comprehensively consider the sample type, research objectives, and available resources.

It is important to note that many of the performance metrics summarized above—such as improvements in gene detection and reductions in diffusion effects—are derived from method-specific datasets and proprietary analytical pipelines. Because these results are generated under varying conditions, including different tissue types, sequencing depths, and preprocessing workflows, direct quantitative comparisons across platforms are inherently limited. For example, reported measures of capture efficiency or diffusion can differ significantly depending on how each method defines key terms such as transcript recovery or signal spillover.

Consequently, the values presented in Table should be interpreted primarily as indicators of within-platform performance rather than definitive cross-platform rankings. To facilitate more rigorous and equitable evaluations, the spatial transcriptomics community would benefit from adopting standardized benchmarking protocols, including the use of shared reference tissues and harmonized pre-processing pipelines.

## 5. Optimization Strategies for Enhancing the Capture Efficiency of FFPE Samples

As the primary component of clinical biobanks, spatial transcriptome analysis of FFPE samples holds significant scientific and clinical value. However, severe RNA degradation and chemical modifications in FFPE samples pose a particular challenge to capture efficiency. To address this issue, several targeted optimization strategies have been developed.

The tolerance to sample degradation varies significantly across different platforms. Stereo-seq V2 (DV200 ≥ 18%) and Xenium (DV200 ≥ 30%) demonstrate the best performance. However, MERFISH 2.0 (DV200 ≥ 50%) and Decoder-seq (DV200 ≥ 35%) require further optimization of their sample processing protocols.

### 5.1. Molecular Compatibility Optimization

RNA in FFPE samples is fragmented due to formaldehyde cross-linking, rendering the traditional poly(T) capture strategy inefficient. Stereo-seq V2 employs random primers instead of poly(T) primers to enable unbiased capture of fragmented RNA. Random primers can bind to any position on the RNA, regardless of the integrity of the poly(A) tail, significantly improving the capture efficiency of FFPE samples. Additionally, a dedicated preprocessing step is crucial for enhancing capture efficiency. Stereo-seq V2 incorporates deparaffinization, rehydration, and decrosslinking procedures, which help reverse some of the damage caused by formaldehyde fixation. BGI’s FFPE V1.1 solution improves gene capture efficiency by 50–200% through optimized probe design and biochemical reaction conditions. These enhancements enable the technology to generate high-quality spatiotemporal omics data from highly degraded clinical samples.

For FFPE tissue in Visium, sample sections first hybridize with pre-designed probes to complete the ligation reaction. Then, the ligation probes are permeated to release their binding to the probes on the slide, thereby capturing gene expression information. Visium FFPE currently designs probes based on human (18,000 genes) and mouse (20,000 genes) gene databases. Using RNA template ligation (RTL), probe pairs specifically target genes in the protein-coding transcriptome, hybridizing with their gene targets and then ligating to each other. Permeation of the tissue releases the ligated probe pairs, allowing them to hybridize with the capture probes on the slide, thus capturing gene expression information.

### 5.2. Signal Diffusion and Control

RNA diffusion resulting from the long-term preservation of FFPE samples is a major factor contributing to the distortion of spatial information. To address this challenge, the BGI FFPE V1.1 solution enhances spatial positioning accuracy to the nanometer scale by optimizing chip probes and experimental protocols. This improvement minimizes signal diffusion both within and outside the tissue, ensuring precise spatial localization, which is essential for studying tumor microenvironments and intercellular interactions.

Although Slide-seq and PIXEL-seq show less molecular diffusion distance, Stereo-seq V2 reduces the molecular diffusion distance (LWHM) by 50%, significantly enhancing spatial resolution in FFPE sample. This improvement enables the technology to analyze the diversity of B-cell receptor clones around the infection site in Mycobacterium tuberculosis infection models with precision and to identify the spatial gradient of decreasing mutation frequency with distance [46].

### 5.3. The Data Utilization Rate Has Improved

To address the high proportion of invalid sequences in FFPE sample sequencing data, BGI’s FFPE V1.1 solution doubled the utilization rate of effective data by optimizing probe design and developing a bioinformatics analysis tool. This optimization significantly reduces the loss of invalid sequencing data (e.g., invalid CID sequences and rRNA sequences), thereby improving the economic efficiency of research. Additionally, it enables large-scale screening of clinical samples.

In the analysis of triple-negative breast cancer FFPE samples, Stereo-seq V2 demonstrated a clear advantage in achieving comprehensive transcriptome coverage. Using spatial inferCNV analysis, researchers identified two tumor subtypes within the samples and detected amplification of the *ESR1* locus. Furthermore, the full-gene coverage enabled detailed alternative splicing analysis, revealing splicing events unique to tumor regions. These analytical capabilities significantly enhance the scientific research value of FFPE samples.

### 5.4. Mixed Sample Testing

The batch effect problem in FFPE sample research can be addressed through the mixed testing compatibility of the innovative FFPE V1.1 solution. This approach allows for the sequencing of FF and FFPE samples within the same batch. By optimizing the library construction kit and protocol, researchers can simultaneously detect spatial-temporal libraries from different sources on a single sequencing chip. This significantly enhances experimental flexibility and sequencing efficiency, facilitating more convenient matching studies.

Additionally, this technology supports multi-tissue splicing analysis. For example, in breast cancer research, FFPE samples from different biopsy sites can be combined on the same chip, significantly enhancing research efficiency. This high-throughput capability enables spatial transcriptome analysis of large cohorts within a single experiment, thereby strongly advancing the development of clinical retrospective studies.

## 6. Future Outlook

Although significant progress has been made in spatial transcriptomics technology, several technical challenges remain. Limited capture efficiency is one of the primary bottlenecks; as resolution increases and the capture/encoding areas become smaller, this issue becomes more pronounced. The mRNA capture efficiency of most current technologies is significantly lower than that of conventional single-cell RNA sequencing. Consequently, more replicates and greater sequencing depth are required to obtain reliable data.

It should be noted that the values presented in Tables should be interpreted primarily as indicators of within-platform performance rather than definitive cross-platform rankings. To enable more rigorous evaluation, the spatial transcriptomics community would benefit from standardized benchmarking protocols based on shared reference tissues and harmonized preprocessing pipelines. Such protocols would facilitate fair comparisons and enhance reproducibility across both academic laboratories and commercial platforms.

Balancing sensitivity and specificity present a significant challenge. Imaging-based methods offer high resolution but are often constrained by detection sensitivity, necessitating a sufficiently high copy number of RNA molecules and multiple imaging capabilities. Conversely, sequencing-based methods can detect low-abundance transcripts but are limited by spatial resolution and diffusion artifacts. Key strategies to achieve this balance include enhancing probe affinity, optimizing the signal amplification system, and reducing background noise. Future advancements may exhibit the following trends:

Firstly, multimodal integration will become a key area of focus. An integrated solution combining nanomaterials, microfluidic technology, and molecular biology will more comprehensively address the bottleneck of capture efficiency. For example, combining the 3D nanostructure of Decoder-seq with the random primer strategy of Stereo-seq V2 is expected to yield a new platform that offers both high sensitivity and full transcriptome coverage. In addition, we may see a growing synergy between transcriptome-wide sequencing and in situ imaging technologies. Meanwhile, the complementary integration of computational prediction methods and experimental techniques, as demonstrated by KAFSTExp, will provide a novel approach to overcoming the efficiency limitations of physical capture.

Secondly, standardization and automation are essential for promoting the widespread adoption of technology [46]. Currently, different platforms use varying standards to evaluate capture efficiency, which complicates cross-study comparisons. Establishing unified efficiency assessment metrics and standardized procedures will facilitate technological optimization, dissemination, and application. Automated processes can reduce human error, improve the reproducibility of results, and facilitate the adoption of technology by biological and clinical researchers.

Thirdly, clinical transformation and application will be a crucial area of development [81]. As compatibility with FFPE samples improves, spatial transcriptomics technology will play an increasingly significant role in precision medicine. In the future, more rapid and cost-effective clinical testing solutions are expected to emerge, supporting tumor diagnosis, prognosis assessment, and treatment decision-making. In fields such as immune microenvironment analysis, tumor heterogeneity research, and host–pathogen interactions, spatial transcriptomics techniques with high capture efficiency are anticipated to provide novel biological insights.

Finally, three-dimensional spatial transcriptomics technology will continue to advance [71]. Currently, most techniques are limited to two-dimensional slice analysis, which makes it difficult to capture the complete spatial architecture of tissues. Although technologies such as MAGIC-seq have demonstrated the potential to reconstruct tissues in three dimensions, achieving high-throughput, high-resolution three-dimensional spatial transcriptomics analysis still requires further technological innovation. These advancements will facilitate a more comprehensive understanding of tissue organization and function.

In conclusion, enhancing RNA capture efficiency remains the pivotal challenge for the advancement of spatial transcriptomics. Addressing this requires concerted, multidisciplinary innovation and collaboration. We are confident that such efforts will yield substantial breakthroughs in the coming years, ultimately transforming spatial transcriptomics from a specialized technique into a routine and indispensable tool for life sciences and medical research.

## Figures and Tables

**Table 1:** Comparison of the capture efficiencies of different spatial transcriptome technologies.

Technology Type	Working Principle	Advantage	Limitations of Capture Efficiency
Spatial barcode array [15,33]	Tissue sections were placed on oligonucleotide arrays with spatial barcodes	High throughput and no bias	Limited by the density and affinity of the probe
In situ sequencing [32]	Amplification and sequencing were performed in situ on tissue sections	High-resolution and single-cell level	The sensitivity is limited and the quantitative accuracy is low
FISH-based [34,35,36,37]	The fluorescent probe hybridizes with the target RNA	High sensitivity, single-molecule detection	It is limited to preset genes and has a limited parallel detection capability
Microneedle capture [38,39,40]	Sample RNA was collected from a specific location using microneedles	Flexible positioning, capable of targeted sampling	Low throughput and limited coverage

## Data Availability

No new data were created or analyzed in this study. Data sharing is not applicable to this article.

## Abbreviations

The following abbreviations are used in this manuscript:

FFPE	formalin-fixed paraffin-embedded
FISH	Fluorescence in situ hybridization
UMI	unique molecular identifier
DNB	DNA nanosphere, nanoball
PCC	Pearson correlation coefficient
FF	fresh frozen
LWHM	left-width at half-maximum
CNV	Copy Number Variation
